# Global research trends in regulating gut microbiome to improve type 2 diabetes mellitus: bibliometrics and visual analysis

**DOI:** 10.3389/fendo.2024.1401070

**Published:** 2024-06-03

**Authors:** Rongsheng Jiang, Zhengri Cong, Likun Zheng, Long Zhang, Qifan Guan, Sixian Wang, Jinxu Fang, Jiahao Chen, Mingjun Liu

**Affiliations:** ^1^ College of Acupuncture and Tuina, Changchun University of Chinese Medicine, Changchun, China; ^2^ College of Medical Information, Changchun University of Chinese Medicine, Changchun, China

**Keywords:** gut microbiome, type 2 diabetes mellitus, research hotspot, research trend, bibliometric analysis

## Abstract

**Background:**

Gut microbiome (GM) and type 2 diabetes mellitus (T2DM) have two-way effects. Improving T2DM by modulating GM in various ways, such as diet, exercise, and medication, is gradually becoming popular, and related studies have yielded positive results. However, there is still a lack of high-quality bibliometric analyses of research in this area. This study aims to systematize and comprehensively summarize the knowledge structure, research tropics, and research trends of GM and T2DM through bibliometric analysis.

**Methods:**

Publications related to GM and T2DM before January 9, 2024, in the Web of Science Core Collection (WOSCC) were searched in this study. Microsoft Excel 2019 was used to analyze publishing trends and CiteSpace (v.6.1.R6 Advanced) was used to analyze institutions, cited journals, references, and keywords.SCImago Graphica (v.1.0.39) was used to analyze countries/regions, institutions’ collaborations, cited authors, and published journals.

**Results:**

We finally included 1004 articles published from 2008 to 2023. The number of published articles showed an upward trend and reached its peak in 2022. China is the country with the largest number of articles, Univ Copenhagen is the institution with the largest number of articles, Fukui, Michiaki, Hamaguchi, Masahide are the scholars with the largest number of articles, and Cani and Patrice D. are the scholars with the largest number of citations. NUTRIENTS(Q1/5.9) published the most publications, while Nature (Q1/64.8; Cited 804 times) is the most frequently cited journal. Gut microbiota, Obesity, and insulin resistance are the most frequently used keywords. This study found that current researches focus on the effects of diet, exercise, and pharmacological modification of GM to improve T2DM and explores specific mechanisms. Future researches will focus on three areas: complications of T2DM and specific physiological processes, methods and measures to regulate GM, and new experimental techniques and assays.

**Conclusion:**

The current researches confirmed the effects and specific mechanisms of modulating GM to improve T2DM. Further exploration of the effects of modulating GM on T2DM complications and specific physiologic processes is a future trend of research. Exploring specific methods for regulating GM and developing new experimental techniques and assays are important for future research.

## 1 Introduction

T2DM is a common chronic disease characterized by hyperglycemia, insufficient insulin secretion, and insulin resistance (1, 2). T2DM accounts for up to 90% of all diabetic patients (3), and often leads to a wide range of comorbidities such as retinopathy, neuropathy, renal damage, and cardiovascular disease (4, 5). T2DM and its complications place an enormous burden on the global health system. In 2013, diabetes was identified as the ninth leading cause of shortened life expectancy globally. In 2015, approximately 5 million people died as a result of T2DM and its complications (6). In recent years, with changing lifestyles and diets, T2DM has become a pandemic worldwide. Data from the International Diabetes Federation show that diabetes will have an impact on the health of 540 million people by 2030 (7).

Combined with recent studies, we found that there is a strong association between GM and obesity & T2DM. Regulating GM has a positive impact on the treatment of T2DM (8–11). GM disorders are typical of patients with T2DM. This phenomenon interferes with the digestion and absorption of fats and carbohydrates and exacerbates disturbances in glucose and lipid metabolism. Adverse alterations in GM derivatives also exacerbate the symptoms of T2DM, such as a decrease in short-chain fatty acids (SCFAs) and bile acids (BAs), and an increase in lipopolysaccharides, exacerbating inflammation and insulin resistance (12, 13). As early as 2008, researchers have found that changes in GM can control intestinal permeability and metabolic endotoxemia in high-fat-fed mice, a phenomenon that can have an impact on inflammation in obesity and diabetes (14) and that pharmacological interventions can also improve glucose tolerance in mice by modulating GM (15). Several subsequent studies have further confirmed the interaction between GM and T2DM (16, 17), and more and more scholars have begun to intervene with GM as a therapeutic target for T2DM, and a series of research results have been achieved (18, 19). It has been found that the longer duration of T2DM may lead to a decrease in the abundance of Bacteroides anthropophilus in the gut, which in turn down-regulates the expression of GLP-1 and leads to an increase in blood glucose (20, 21). Reduced abundance of Mycobacterium avium and Mycobacterium faecalis may induce an inflammatory response by triggering the RLR signaling pathway and activating the downstream NF-κB signaling pathway (21). The results of clinical trial showed that administration of Gegen Qinlian decoction improved GM homeostasis and increased the abundance of Faecalibacterium, which in turn improved the metabolic disorders and inflammatory state in T2DM patients (22). A recent study noted that polysaccharides from Phellinus linteus promote GLP-1 secretion, increase insulin release, and lower blood glucose by regulating SCFAs and Bas metabolism (23). In conclusion, current studies have identified the restoration of GM homeostatic balance by regulating the number and abundance of each genus of GM and thus improving the metabolism of related derivatives as an effective measure for the treatment of T2DM (24–26).

Currently, studies of GM and T2DM are becoming more common. However, there is still a lack of organizing and summarizing the current research hotspots and future research trends in the field. Bibliometric analysis allows quantitative analysis and summarization of relevant publications in a research area using information visualization methods (27). This will allow us to visualize the knowledge structure of the field and identify current research frontiers and hotspots (28). The aim of this study was to use bibliometric analysis to comprehensively and systematically organize the literature related to the modulation of GM for the treatment of T2DM, and to provide references and directions for future research in this field.

## 2 Materials and methods

### 2.1 Data sources and search strategy

We collected literatures from the WOSCC database and conducted a search of the literatures on January 9, 2024. To improve the reliability and representativeness of the literatures, we used the MeSH terms provided by Pubmed as the keywords for the search.The specific search strategy is:TS=(“Gastrointestinal Microbiome*” OR “Microbiome, Gastrointestinal” OR “Gut Microbiome*” OR “Microbiome, Gut” OR “Gut Microflora” OR “Microflora, Gut” OR “Gut Microbiota*” OR “Microbiota, Gut” OR “Gastrointestinal Flora” OR “Flora, Gastrointestinal” OR “Gut Flora” OR “Flora, Gut” OR “Gastrointestinal Microbiota*” OR “Microbiota, Gastrointestinal” OR “Gastrointestinal Microbial Community*” OR “Microbial Community, Gastrointestinal” OR “Gastrointestinal Microflora” OR “Microflora, Gastrointestinal” OR “Gastric Microbiome*” OR “Microbiome, Gastric” OR “Intestinal Microbiome*” OR “Microbiome, Intestinal” OR “Intestinal Microbiota*” OR “Microbiota, Intestinal” OR “Intestinal Microflora” OR “Microflora, Intestinal” OR “Intestinal Flora” OR “Flora, Intestinal”) AND (“Diabetes Mellitus, Type 2” OR “Diabetes Mellitus, Non Insulin Dependent” OR “Stable Diabetes Mellitus” OR “Diabetes Mellitus, Type II” OR “NIDDM” OR “Diabetes Mellitus, Noninsulin Dependent” OR “Type 2 Diabetes Mellitus” OR “Noninsulin Dependent Diabetes Mellitus” OR “Type 2 Diabetes” OR “Diabetes, Type 2”).

### 2.2 Data processing and analysis

**Figure 1:**
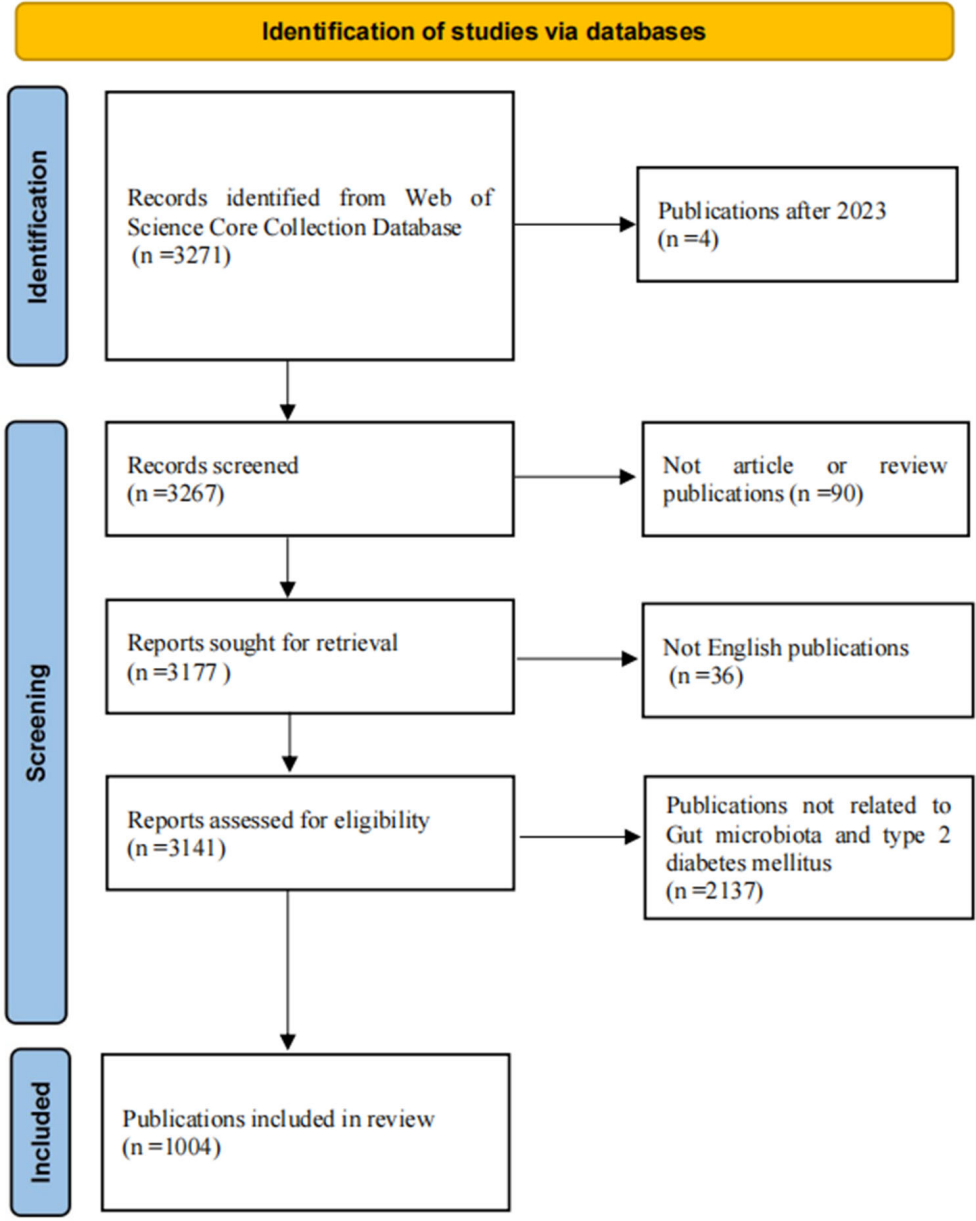
Schematic of data collection and screening.

To ensure the accuracy of the results of the data analysis, the retrieved publications were screened, first by excluding duplicates through EndNote X9.3.3 software, and then by manually reading the titles and abstracts to screen for publications that met the inclusion criteria. This process was carried out independently by 2 researchers (CZR and GQF), and when disagreements arose, the decision was made by a third researcher (FJX). The inclusion criteria for publications were (1) The types of publications were ARTICLE or REVIEW.(2) English language publications. (3) Study species were not restricted and both animal models and human studies could be included.The exclusion criteria for publications were (1) Publications that were not relevant to the topic of the study. (2) Publications for which full text was not available. Since there were fewer publications from 2024 to form convincing results, we included only publications from 2023 and earlier.We finally screened 1004 publications, including 780 publications of Article type and 224 publications of Review type. **Figure 1** illustrates the specific screening process. The data analysis of the screened publications was performed by a researcher (CJH) who independently operated the software.

Microsoft Excel 2019, CiteSpace (v.6.1.R6 Advanced) and SCImago Graphica (v.1.0.39) were applied to analyze the data. Microsoft Excel 2019 exported the trend of annual number of publications; CiteSpace (v.6.1.R6 Advanced) was used to analyze institution co-occurrence, cited journals, dual map overlay of journals, references, keywords; SCImago Graphica (v.1. 0.39) was used to analyze countries/regions, institution clustering, cited authors, and published journals.

The parameters of CiteSpace (v.6.1.R6 Advanced) software were set as follows: Time Span: 2008-2023; Slice Length: 1 year; Selection Criteria: gindex(k=25); Pruning: pathfinder, pruningsliced networks. after the setup was completed, authors, institutions, and keywords were selected as network nodes for visualization and analysis, respectively.The main parameters of SCImago Graphica (v.1.0.39) software are set as follows: Size: frequency; Color:clusters; Lable: choose according to the content of the analysis, Use the same color as marks; Layout: Circular; Edges: Use the same color as marks.

## 3 Results

### 3.1 Trends in the growth of publications

**Figure 2:**
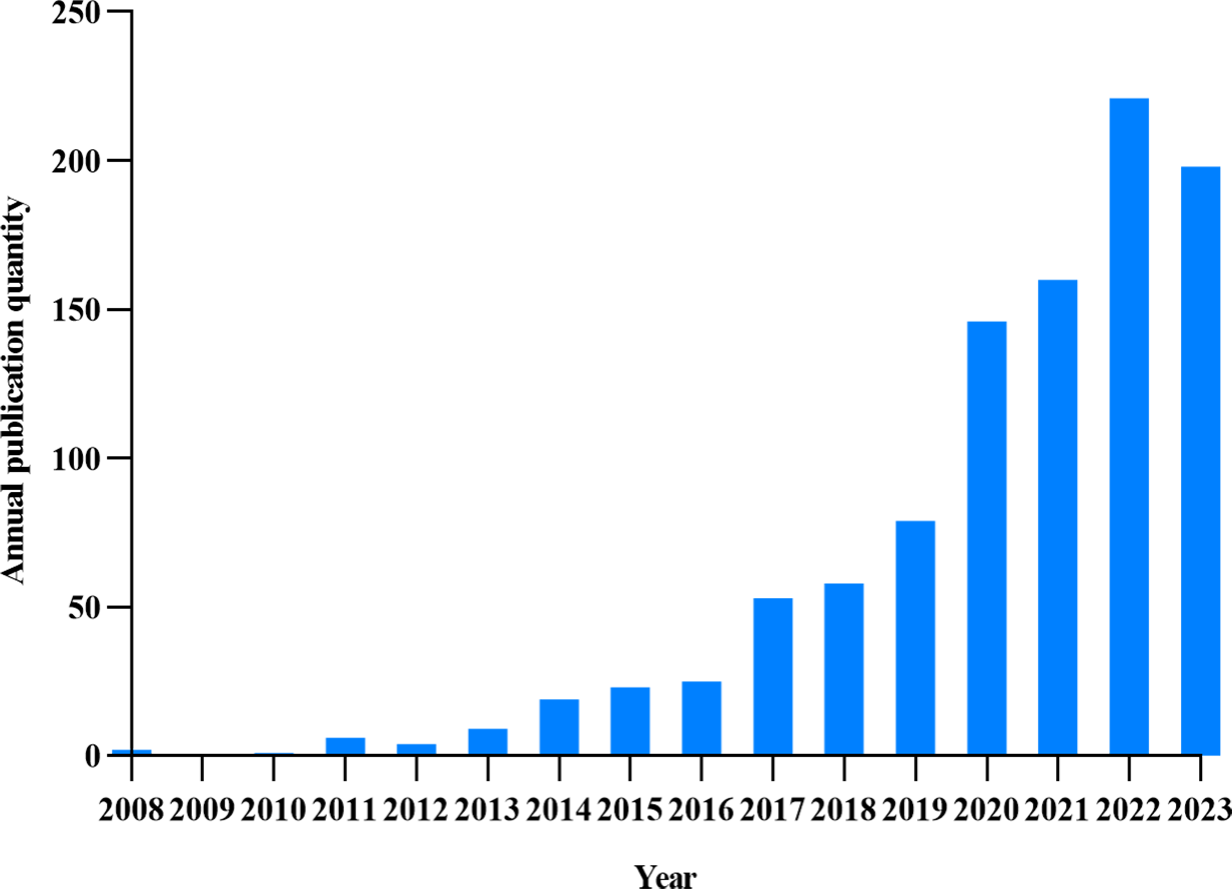
Trends in the posting of papers on the relationship between GM and T2DM.

**Figure 2** illustrates the annual volume of publications. 1004 publications were published between 2008 and 2023, and the number of publications showed an upward trend. The number of publications exceeded 50 in 2017 and 150 in 2020. 2022 had the highest number of publications with more than 200.The analysis of the number of publications shows that the current number of publications is in a relatively stable state. This theme continues to attract the attention of researchers.

### 3.2 Countries/Regions distribution and cooperation

**Figure 3:**
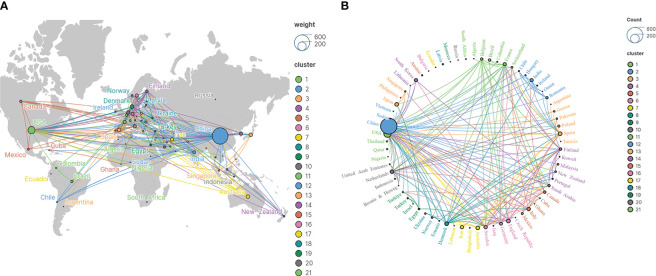
Country/region visual analysis. **(A)** Country/region world distribution. **(B)** Country/region cooperation analysis.

A total of 187 countries/regions contributed to the publications. **Table 1** shows the top 15 countries in terms of the number of publications, none of which has published less than 20 publications. **Figure 3A** illustrates the co-occurrence relationship among these countries. The results show that the studies’ citations related to this theme are widely popular around the world. China and the United States are the main contributors with 571 and 118 publications respectively, far ahead of other countries/regions. It can be seen that China and the United States have invested a lot of money and energy in conducting relevant research. This may be due to the adverse human health and socio-economic effects caused by the widespread prevalence of T2DM in the two countries.

**Table 1:** Top 15 countries/regions in terms of number of articles published.

Rank	Count	Country/regions
1	571	CHINA
2	118	USA
3	33	DENMARK
4	32	SPAIN
5	31	JAPAN
6	30	AUSTRALIA
7	29	NETHERLANDS
8	28	ITALY
9	25	FRANCE
10	25	ENGLAND
11	25	IRAN
12	25	SWEDEN
13	24	INDIA
14	24	SOUTH KOREA
15	20	GERMANY

**Figure 3B** illustrates the cooperative relationships of relevant countries/regions worldwide, which can be categorized into 21 clusters, indicating that a wide range of cooperative relationships exist among these countries. The cooperative relationship between two clusters, China-UK-Germany-Sweden and USA-India-Romania, is more prominent. France and Japan also have cooperative relationships with several countries respectively. It can be seen that cooperation between countries/regions has promoted the development of researches.

### 3.3 Distribution and cooperation between institutions

**Figure 4:**
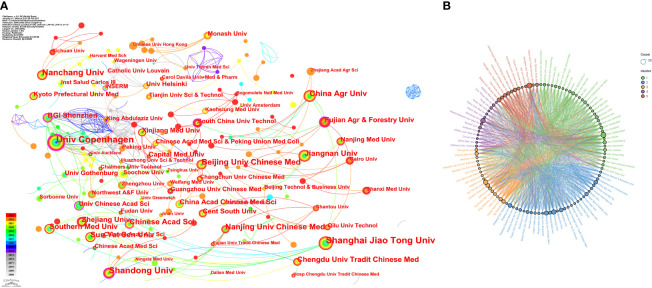
Visual analysis of the organization. **(A)** Co-occurrence network view of institutions. **(B)** Cluster analysis network view of the organization.

A total of 3683 institutions contributed to publications on this topic. **Table 2** shows the top 15 institutions in terms of publication volume. 15 institutions have no less than 12 publications.The number of publications of Univ Copenhagen is 27, the centrality is 0.21, and the total link strength is 49, indicating that this organization has an important position in this field of research.The number of publications of Shanghai Jiao Tong Univ is also 27, with a centrality of 0.09 and a total link strength of 40, indicating that this organization has also achieved a great deal of researches and has greater influence. In addition, Chinese Acad Sci achieved a total link strength of 42, indicating that this organization has more cooperation with other organizations. **Figure 4A** illustrates the network view of co-occurrence analysis for these institutions. **Figure 4B** illustrates the network view of cluster analysis for these institutions. These institutions are divided into 5 cooperative clusters, and there is close cooperation between the organizations in each cluster, and there is also obvious cooperation between the clusters.

**Table 2:** Top 15 institutions in terms of number of articles published.

Rank	Count	Centrality	Institutions	Total link strength
1	27	0.21	Univ Copenhagen	49
2	27	0.09	Shanghai Jiao Tong Univ	40
3	22	0.1	Shandong Univ	28
4	21	0.03	Nanchang Univ	7
5	18	0.04	Beijing Univ Chinese Med	23
6	16	0.03	Nanjing Univ Chinese Med	7
7	16	0.03	China Agr Univ	14
8	15	0.17	Chinese Acad Sci	42
9	15	0.02	Jiangnan Univ	13
10	15	0.01	Sun Yat Sen Univ	33
11	14	0.02	Southern Med Univ	29
12	14	0.01	Chengdu Univ Tradit Chinese Med	6
13	13	0.39	BGI Shenzhen	40
14	13	0	Zhejiang Univ	6
15	12	0.13	Fujian Agr & Forestry Univ	11

### 3.4 Authors’ publications analysis and collaborations

**Figure 5:**
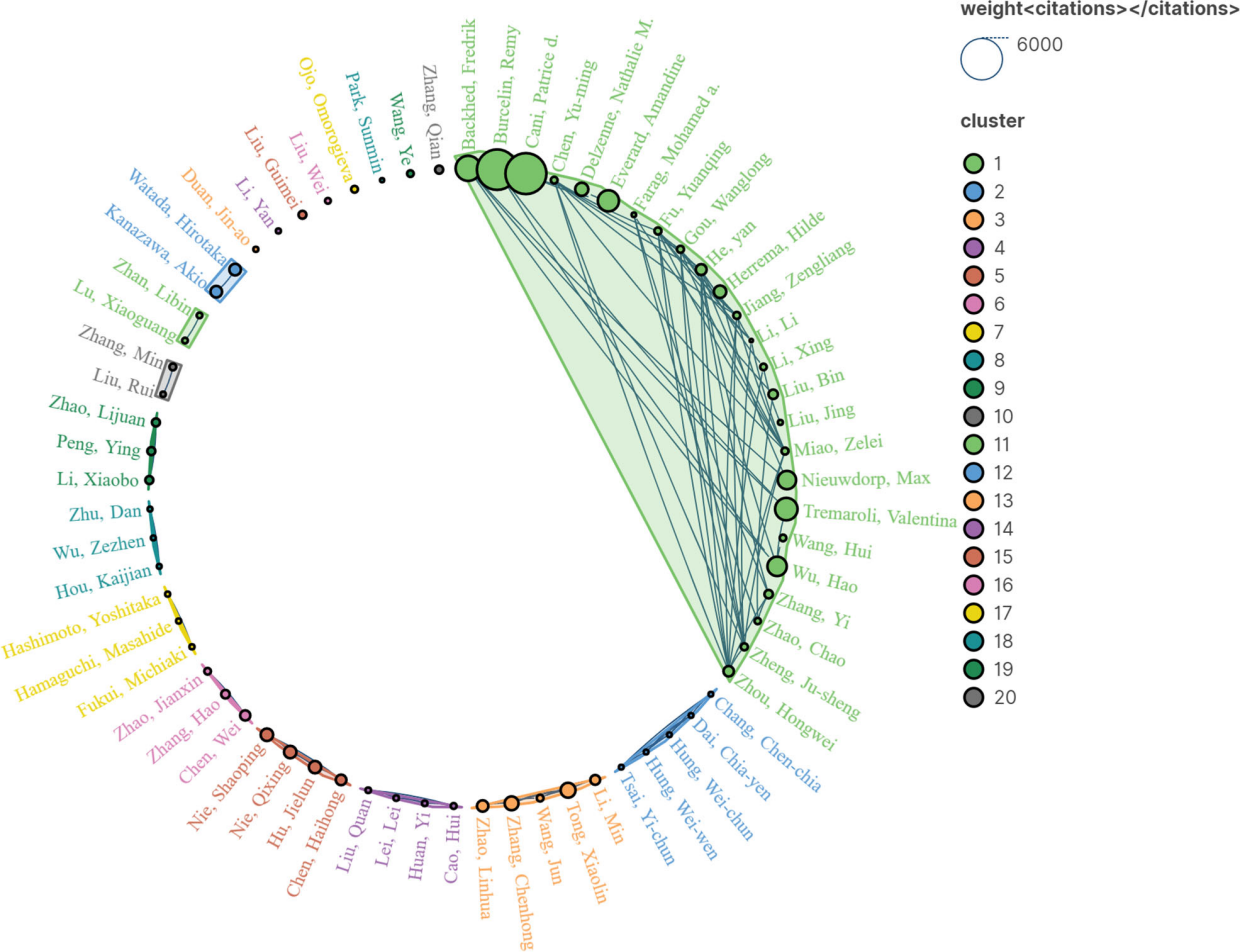
Network view of cited authors.

A total of 6425 authors have contributed to publications in the field. **Table 3** demonstrates the authors who are in the top 15 in terms of the amount of published literature. Fukui Michiaki and Hamaguchi Masahide both published 8 articles, placing them in the top 2. Backhed, Fredrik’s centrality of 0.03 ranks first. **Table 4** demonstrates the top 15 authors with the highest number of citations to their publications, with Cani, Patrice’s article has the highest number of citations at 5172. **Figure 5** presents a cluster analysis network view of the highly cited authors, which form 20 distinct clusters with stable collaborations between several authors. This suggests that studies in this field have formed research teams in the course of its development.

**Table 3:** Top 15 authors in terms of number of publications.

Rank	Count	Centrality	Authors
1	8	0	Fukui, Michiaki
2	8	0	Hamaguchi, Masahide
3	7	0.01	Burcelin, Remy
4	7	0	Cani, Patrice D
5	7	0	Hu, Jielun
6	7	0	Liu, Bin
7	7	0	Nie, Qixing
8	7	0	Nie, Shaoping
9	6	0.03	Backhed, Fredrik
10	6	0.01	Nielsen, Trine
11	6	0	Chen, Wei
12	6	0	Hashimoto, Yoshitaka
13	6	0	Li, Xing
14	5	0.02	Hansen, Torben
15	5	0.01	Farag, Mohamed A

**Table 4:** Top 15 authors with cited papers.

Rank	Authors	Citations	Total link strength
1	Cani, Patrice d.	5172	10
2	Burcelin, Remy	5049	4
3	Backhed, Fredrik	1946	8
4	Tremaroli, Valentina	1560	7
5	Everard, Amandine	1375	8
6	Wu, Hao	1153	7
7	Nieuwdorp, Max	1031	5
8	Tong, Xiaolin	681	12
9	Zhang, Chenhong	591	4
10	Delzenne, Nathalie m.	539	7
11	Nie, Shaoping	487	21
12	Nie, Qixing	481	21
13	Herrema, Hilde	481	4
14	Hu, Jielun	472	19
15	Kanazawa, Akio	419	5

### 3.5 Analysis of journals and cited journals

**Figure 6:**
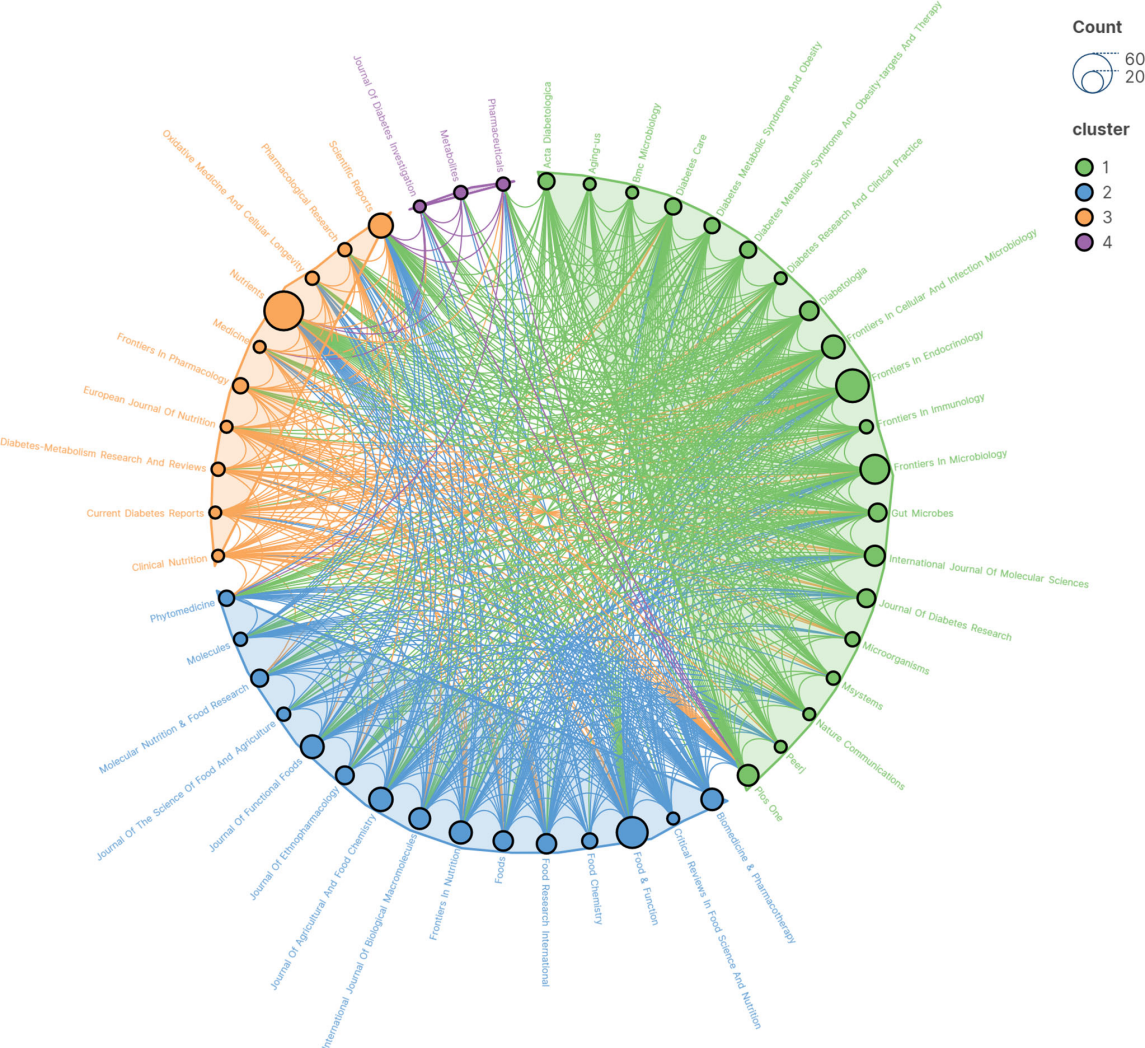
Network view of issuing journals.

A total of 318 journals published publications related to this topic. **Table 5** demonstrates the 15 journals with the highest number of publications. The 15 journals published a total of 328 publications which represents 32.67% of all publications. INTERNATIONAL JOURNAL OF BIOLOGICAL MACROMOLECULES has the highest impact factor of 8.2. 10 of the 15 journals are in Q1 and 5 are in Q2. **Figure 6** illustrates a network view of the issuing journals, with a larger area of nodes indicating more issues. These journals can be clustered into 4 clusters and there is a correlation between journals citing each other.

**Table 5:** Top 15 journals in terms of publications.

Rank	Journalss	Count	Division/IF
1	NUTRIENTS	51	Q1/5.9
2	FRONTIERS IN ENDOCRINOLOGY	37	Q2/5.2
3	FOOD & FUNCTION	33	Q1/6.1
4	FRONTIERS IN MICROBIOLOGY	29	Q2/5.2
5	SCIENTIFIC REPORTS	20	Q2/4.6
6	JOURNAL OF AGRICULTURAL AND FOOD CHEMISTRY	19	Q1/6.2
7	FRONTIERS IN CELLULAR AND INFECTION MICROBIOLOGY	18	Q1/5.7
8	JOURNAL OF FUNCTIONAL FOODS	18	Q2/5.0
9	FRONTIERS IN NUTRITION	17	Q2/5.0
10	BIOMEDICINE & PHARMACOTHERAPY	16	Q1/7.5
11	INTERNATIONAL JOURNAL OF BIOLOGICAL MACROMOLECULES	15	Q1/8.2
12	PLOS ONE	15	Q1/3.7
13	INTERNATIONAL JOURNAL OF MOLECULAR SCIENCES	14	Q1/5.6
14	FOOD RESEARCH INTERNATIONAL	13	Q1/8.1
15	FOODS	13	Q1/5.2

**Figure 7:**
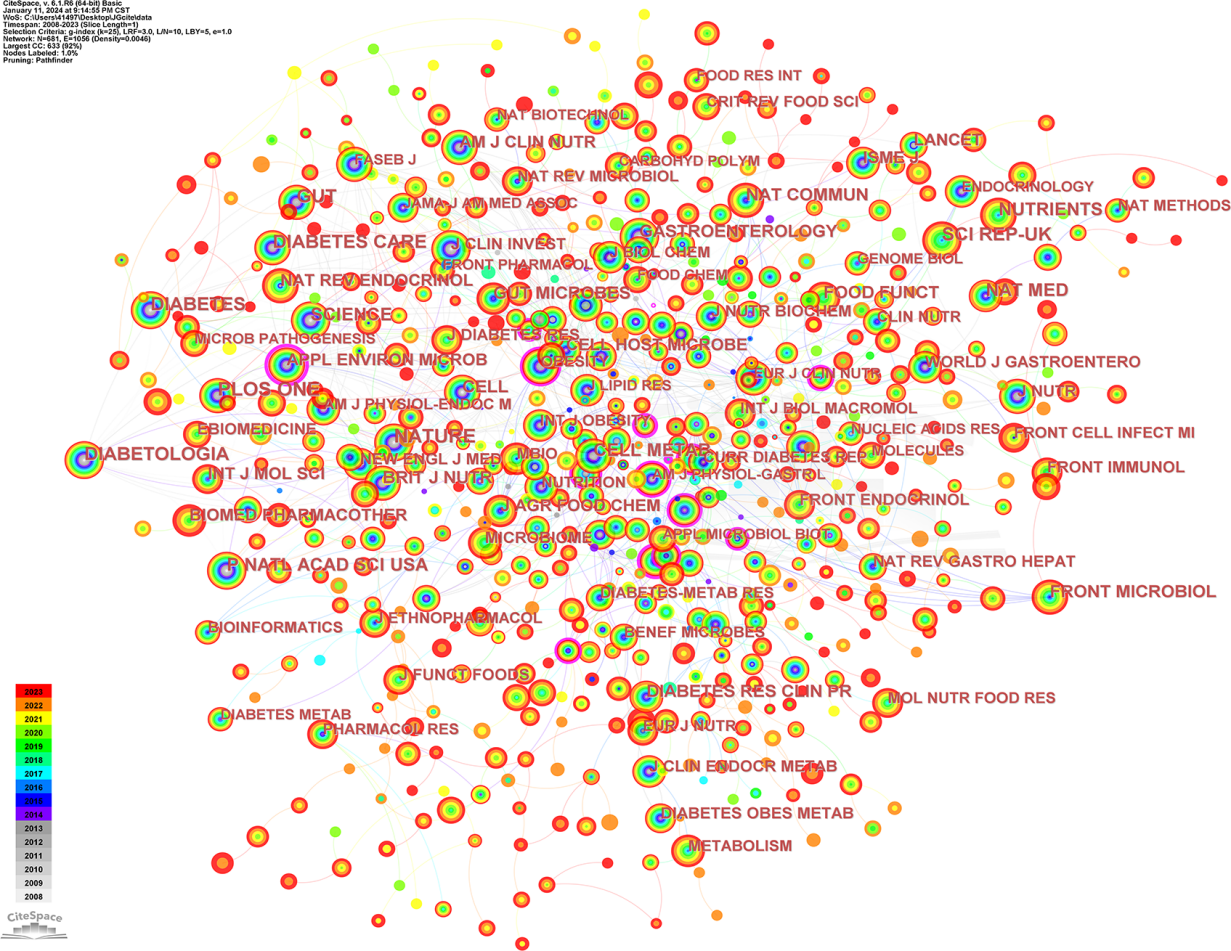
Network co-occurrence view of cited journals.

**Table 6** shows the top 15 most frequently cited journals, and **Figure 7** shows the network analysis view of cited journals. NATURE (Q1/64.8; 804 citations) is the most frequently cited journal. NATURE, PLOS ONE, and DIABETOLOGIA all have a centrality of 0.03, making them the 3 journals with the highest centrality. 13 journals were in Q1 and 2 journals were in Q2. Nat Med had the highest impact factor of 82.9.

**Table 6:** Top 15 most cited journals.

Rank	Count	Centrality	Cited Journal	Division/IF
1	807	0.03	NATURE	Q1/64.8
2	722	0.03	PLOS ONE	Q2/3.7
3	596	0	DIABETES	Q1/7.7
4	586	0	DIABETES CARE	Q1/16.2
5	558	0.02	GUT	Q1/24.5
6	546	0.03	DIABETOLOGIA	Q1/8.2
7	544	0.02	SCI REP-UK	Q2/4.6
8	498	0	SCIENCE	Q1/56.9
9	485	0.02	NUTRIENTS	Q1/5.9
10	468	0	P NATL ACAD SCI USA	Q1/11.1
11	444	0.06	CELL METAB	Q1/29
12	421	0.05	NAT MED	Q1/82.9
13	395	0.01	NAT COMMUN	Q1/16.6
14	378	0	CELL	Q1/64.5
15	373	0.04	FRONT MICROBIOL	Q1/5.2

**Figure 8:**
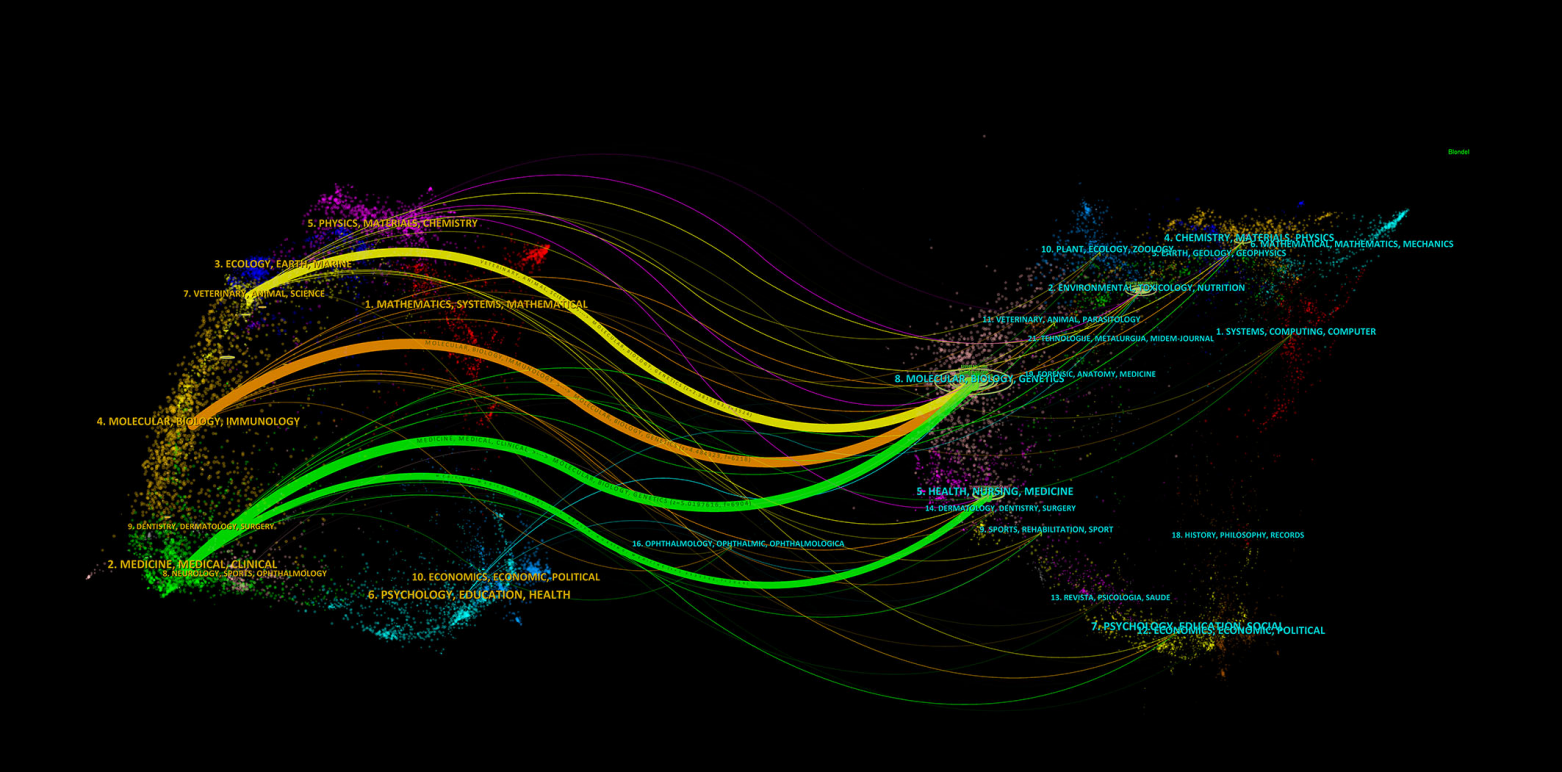
Analyzed view of the bimorphic overlay of the journals.

In **Figure 8**, dual map overlay of journals shows the distribution of topics. The labels indicate the subject topics covered and the colored paths indicate the citation relationships. The results show four significant citation paths. It can be seen that journals under the theme “MOLECULAR,BIOLOGY,GENETICS” are often cited by “VETERINARY,ANIMAL,SCIENCE” “ MOLECULAR, BIOLOGY, IMMUNOLOGY” and “MEDICINE, MEDICAL, CLINICAL”;Journals under the theme “HEALTH, NURSING, MEDICINE” are often cited by journals under the theme “MEDICINE, MEDICAL, CLINICAL”.

### 3.6 Analysis of co-cited references

**Figure 9:**
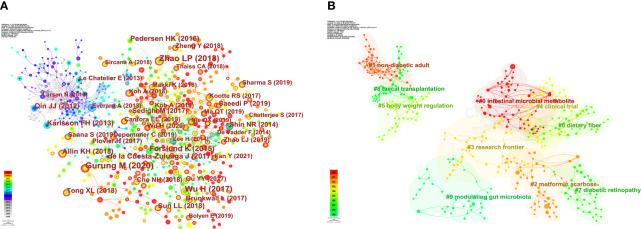
Network analysis view of co-cited references. **(A)** Co-occurrence analysis network view. **(B)** Cluster analysis network view.

Co-citation relationships are formed when multiple documents are cited multiple times by different documents, and such co-citation relationships are often used to determine the degree of association between different documents. **Table 7** demonstrates the top 15 cited references and **Figure 9A** shows the network view of co-occurrence analysis for these references. The most cited reference is “Role of gut microbiota in Type 2 diabetes pathophysiology” by Gurung M published on EBIOMEDICINE in 2020. The next is “Gut bacteria selectively promoted by dietary fibers alleviate type 2 diabetes” by Zhao LP published on SCIENCE in 2018. The third is “Metformin alters the gut microbiome of individuals with treatment-naive type 2 diabetes, contributing to the therapeutic effects of the drug” by Wu H published on NAT MED in 2017. These papers focus on the direct association between GM and T2DM, exploring the physiological links between GM and T2DM the effects of different interventions on GM, and the ameliorative effects on T2DM. **Figure 9B** illustrates the network view of the cluster analysis. The results showed that current research hotspots between GM and T2DM include “intestinal microbial metabolite” “non-diabetic adult” “metformin acarbose” “research frontier” and “clinical trial”. Q= 0.8379 > 0.3 and S = 0.9453 > 0.7, suggesting that this analysis resulted in a significant clustering structure and a convincing conclusion.

**Table 7:** Top 15 most frequently cited references.

Rank	Count	Centrality	Year	Cited Reference
1	177	0	2020	Gurung M, 2020, EBIOMEDICINE, V51, P0, DOI 10.1016/j.ebiom.2019.11.051
2	143	0.01	2018	Zhao LP, 2018, SCIENCE, V359, P1151, DOI 10.1126/science.aao5774
3	141	0.01	2017	Wu H, 2017, NAT MED, V23, P850, DOI 10.1038/nm.4345
4	91	0	2015	Forslund K, 2015, NATURE, V528, P262, DOI 10.1038/nature15766
5	82	0	2017	de la Cuesta-Zuluaga J, 2017, DIABETES CARE, V40, P54, DOI 10.2337/dc16-1324
6	77	0	2013	Karlsson FH, 2013, NATURE, V498, P99, DOI 10.1038/nature12198
7	76	0	2016	Pedersen HK, 2016, NATURE, V535, P376, DOI 10.1038/nature18646
8	69	0	2012	Qin JJ, 2012, NATURE, V490, P55, DOI 10.1038/nature11450
9	67	0	2018	Allin KH, 2018, DIABETOLOGIA, V61, P810, DOI 10.1007/s00125-018-4550-1
10	62	0.02	2018	Sun LL, 2018, NAT MED, V24, P1919, DOI 10.1038/s41591-018-0222-4
11	60	0	2018	Tong XL, 2018, MBIO, V9, P0, DOI 10.1128/mBio.02392-17
12	57	0.01	2019	Saeedi P, 2019, DIABETES RES CLIN PR, V157, P0, DOI 10.1016/j.diabres.2019.107843
13	57	0	2017	Sedighi M, 2017, MICROB PATHOGENESIS, V111, P362, DOI 10.1016/j.micpath.2017.08.038
14	55	0.31	2014	Shin NR, 2014, GUT, V63, P727, DOI 10.1136/gutjnl-2012-303839
15	55	0	2017	Brunkwall L, 2017, DIABETOLOGIA, V60, P943, DOI 10.1007/s00125-017-4278-3

### 3.7 Keywords analysis

#### 3.7.1 Keywords co-occurrence analysis

**Figure 10:**
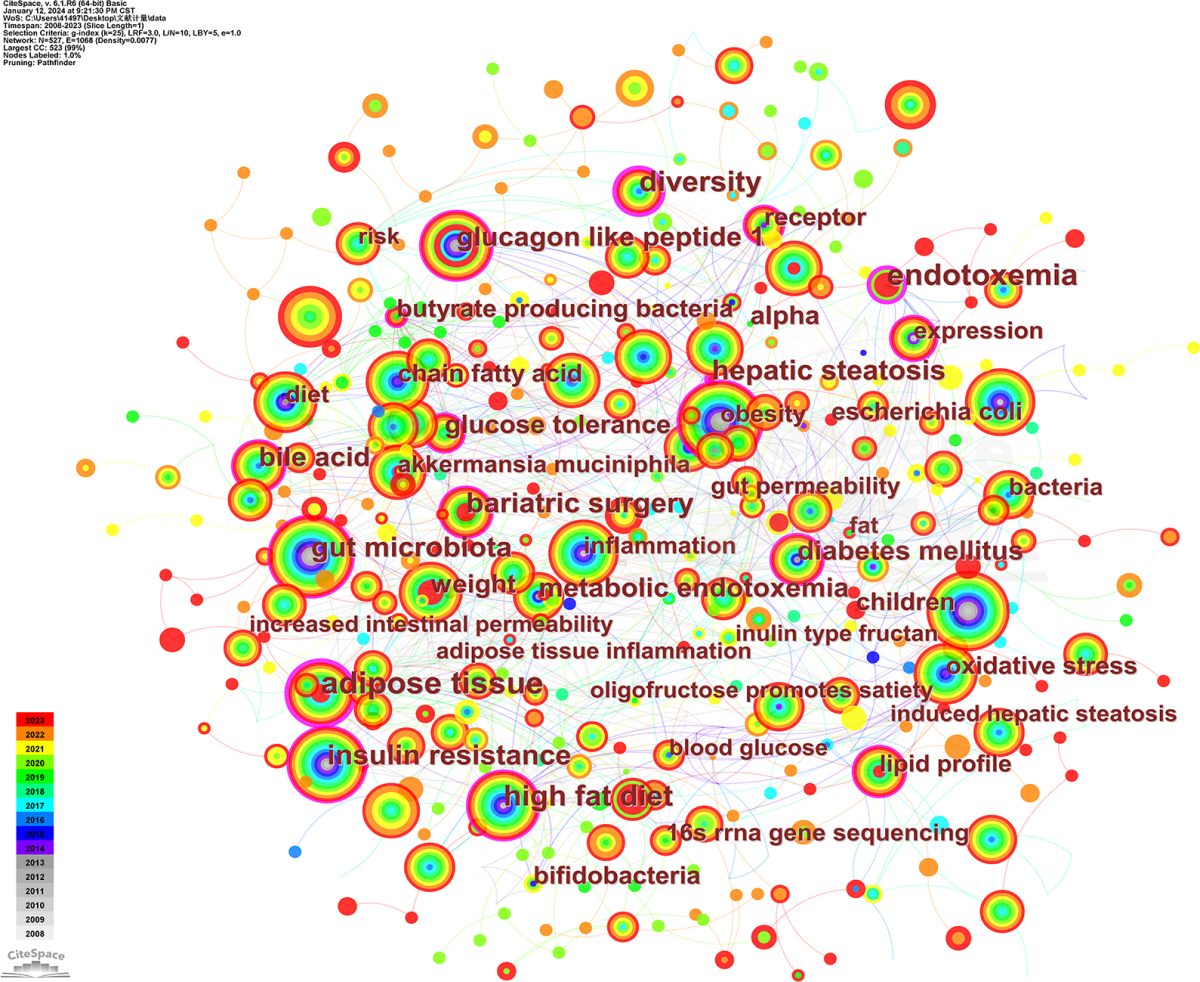
Co-occurrence network view of keywords.

Keywords that appear in the same literature with associations form co-occurrence relationships, and the co-occurrence network view reflects the research focus and connections in this area.A total of 527 keywords were found in 1004 publications. **Table 8** shows the 15 keywords with the highest frequency of occurrence.The most frequent keywords were “gut microbiota”, “type 2 diabetes mellitus”, “obesity”, “insulin resistance” and “inflammation”. “insulin resistance” and “inflammation”, which reflect the current research hotspots in this field.The co-occurrence network view of the keywords is shown in **Figure 10**, and the size of the nodes reflects the frequency of keyword occurrence.

**Table 8:** 15 keywords with the highest frequency of occurrence.

Rank	Count	Centrality	Keywords
1	755	0.12	gut microbiota
2	495	0	type 2 diabetes mellitus
3	387	0.12	obesity
4	238	0.12	insulin resistance
5	170	0.02	inflammation
6	137	0.06	chain fatty acid
7	123	0	metagenome
8	120	0.02	glucose
9	111	0.06	association
10	104	0	metabolism
11	102	0.05	diet
12	83	0.08	oxidative stress
13	81	0	insulin sensitivity
14	79	0.04	mice
15	75	0.04	gut microbiome

#### 3.7.2 Keywords clustering analysis

**Figure 11:**
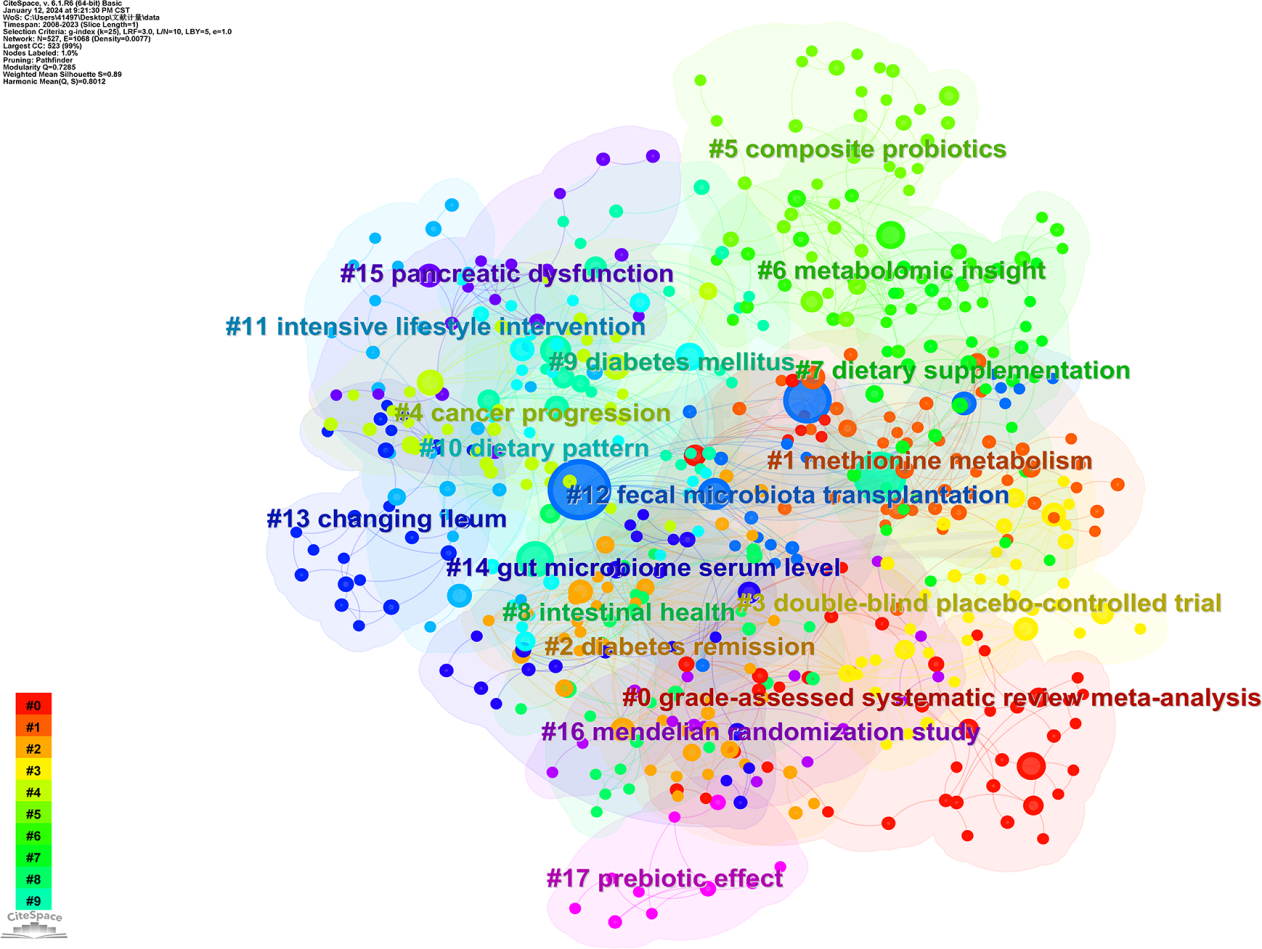
Network view of keywords cluster analysis.

**Figure 11** illustrates the network view of the keyword clustering analysis. The results of the analysis show Q = 0.7285 > 0.3 and S = 0.89 > 0.7, indicating that the clustering structure of this analysis is significant and the results are convincing. The 18 notable clusters that relate to diabetes and related physiological processes are #1 methionine metabolism, #2 diabetes remission, #9 diabetes mellitus, and #15 pancreatic dysfunction. Relate to GM and intervention modalities are #5 composite probiotics, #7 dietary supplementation, #8 intestinal health, #10 dietary pattern, #11 intensive lifestyle intervention, #12 fecal microbiota transplantation, #13 changing ileum, #17 prebiotic effect; Relate to the type of study, experimental technique, and assay were #0 grade-assessed systematic review meta-analysis, #3 double-blind placebo-controlled trial, #6 metabolomic insight, #14 gut microbiome serum level, #16 mendelilan randomization study.

#### 3.7.3 Keywords bursts analysis

**Figure 12:**
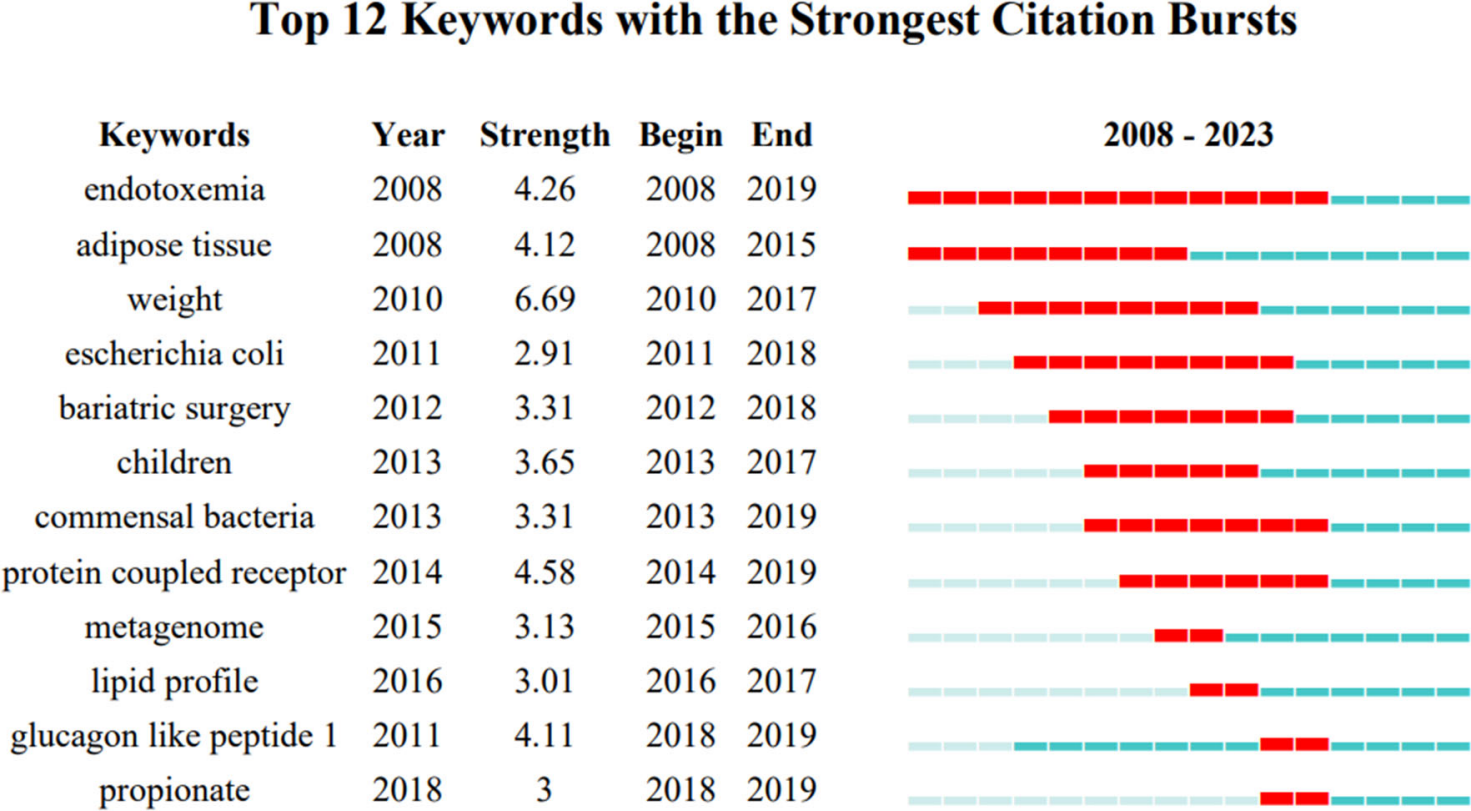
View of keywords bursts analysis.

The keywords burst analysis shows the hotspot changes in the field, and **Figure 12** demonstrates the results of the analysis.”Endotoxemia” and “adipose tissue” were the first two keywords to show up in persistent heat. The most recent keywords to show persistent heat were “glucagon-like peptide” and “propionate”. “Endotoxemia” is the keyword with the longest duration.

#### 3.7.4 Keywords timeline analysis

To further analyze the changes in keywords, we conducted a timeline analysis of the keywords, which is detailed in **Supplementary Figure S1**. “Grade-assessed systematic review meta-analysis” has been a hot research topic since 2014 and is the most prominent cluster. The research hotspot for this cluster is lipid profile. The 2nd most prominent cluster is “methionine metabolism”, which has been a research hotspot since 2011, and its research hotspot is fat. The 3rd most prominent cluster is “diabetes remission”, and research in this cluster focuses on diabetes mellitus.

## 4 Discussion

### 4.1 Overview of research between GM and T2DM

In this study, we conducted a bibliometric analysis by analyzing the relevant literature about GM and T2DM in the WOSCC database. We have mainly reviewed the current research overview in this field from the perspectives of trends in the number of publications, distribution, and cooperation of countries/regions, distribution and cooperation of institutions, number of publications and cooperation of authors, journals, and cited journals, co-cited references, and keywords.

In terms of the number of publications, the overall research output in this field has been on an upward trend since 2008 and will reach its peak in 2022. Although the number of publications declined in 2023, it still maintains a high number of publications, which indicates that the research in this field is still a hotspot and has high research value.

In terms of countries/regions, the 187 countries involved in this area of research are spread across five continents. China and the US are the 2 countries with the largest number of publications. In terms of collaborations, there are closer collaborations between China-UK-Germany-Sweden and US-India-Romania.

In terms of issuing institutions, among the 3683 institutions involved, Univ Copenhagen and Shanghai Jiao Tong Univ were the 2 institutions with the largest number of publications.Univ Copenhagen has the highest centrality among all the institutions and the strongest collaborative relationship with other institutions.

In terms of authors, 6425 authors are involved in the field. The highest number of publications is by Fukui, Michiaki, and Hamaguchi, Masahide, the highest centrality is by Backhed, Fredrik, and the most cited article is by Cani, Patrice d. These authors form distinct clusters, with stable collaborations between them.

In terms of journals, the journal with the highest number of publications was NUTRIENTS (Q1/5.9), and the journal with the highest number of citations was NATURE (Q1/64.8; 804 citations). The top 15 journals in terms of publications totaled 328 publications or 32.67% of all publications. The most centrally cited journals were NATURE, PLOS ONE, and DIABETOLOGIA. The cited journals were mainly distributed in the themes of “MOLECULAR, BIOLOGY, GENETICS” and “HEALTH, NURSING, MEDICINE”.

Analysis of the co-cited references showed that the top 3 cited publications were from Gurung M, Zhao LP, and Wu H. These publications mainly focused on the physiological link between GM and T2DM and the improvement of T2DM by regulating GM.

The analysis of keywords showed that gut microbiota, type 2 diabetes mellitus, Obesity, insulin resistance, and inflammation appeared at the top of the frequency list. This reflects the current research hotspot in this field, obesity and T2DM often occur together, and patients with both diseases suffer from chronic low-level inflammation and insulin resistance, so these conditions should be comprehensively considered in related studies. High-frequency keywords also included GM derivatives and related experimental techniques. The clustering analysis results suggested that evidence-based medical research has received focused attention from scholars, which can provide a research basis and high-quality evidence for research in this field. The bursts analysis revealed that glucagon-like peptides and propionate are two recently emerged hotspots for ongoing research.

### 4.2 Research progress on the link between GM and T2DM

#### 4.2.1 Regulating GM to improve T2DM is gradually becoming popular

GM plays a significant role in the pathogenesis of T2DM (29, 30), GM dysregulation is a common state in patients with T2DM. Bifidobacterium and Mycobacterium spp. are considered to be the most relevant genera to T2DM (31–33). In animal experiments, researchers have found that Bifidobacterium inhibits fat accumulation, reduces obesity, attenuates insulin resistance, lowers blood glucose levels, and improves glucose tolerance (34, 35). Cross-sectional studies have demonstrated a negative association between Bifidobacterium spp. and T2DM (33, 36). However, one study found that the accumulation of an ovine Anopheles strain drove the expansion of IL-17-producing CD27-MAIT cells (37). In addition to Bifidobacterium and Anaplasma spp. other genera have also shown association with T2DM (38). In conclusion, regulating GM will probably be an effective measure to improve T2DM (39–41).

#### 4.2.2 Main pathways of action for regulating GM to improve T2DM

GM improves T2DM by modulating inflammation, maintaining intestinal barrier function, and correcting disorders of glucolipid metabolism.Although lipopolysaccharides as GM derivatives promote endotoxemia and low-level inflammation (42, 43), more microorganisms and their metabolites produce anti-inflammatory factors and chemokines, which can improve inflammation in the organism. Short-chain fatty acids, as the main derivatives of GM, influence the inflammatory state of the organism. Butyric acid modulates macrophage function by inhibiting HDACs and decreases the expression levels of pro-inflammatory mediators NO, IL-6, and IL-12 (44). A 2009 study has confirmed that GM has a positive effect on maintaining intestinal barrier function (45). Probiotic supplementation reduces the expression of TNF-α and IL-6 (46), promotes the expression of ZO-1, and maintains intestinal barrier function (47, 48). Data from a scoping review showed that 40 GM taxa were associated with glucose and 17 with insulin (49). Of these, Enterobacteriaceae were directly associated with fasting and postprandial glucose (50–52). Probiotic supplementation significantly improved glycemic and lipid parameters in mice and also promoted GLP-1 expression by up-regulating the activities of GPR43/41, GCG, and PC1/3 thereby promoting insulin secretion (53). In conclusion, the use of GM as a therapeutic target for T2DM is increasingly supported by combining the results of current clinical studies and animal experiments.

Despite the promising findings of current researches related to GM and T2DM, there are still questions that hinder the further development of related researches. Although researchers have discovered a link between GM and T2DM, and the modulation of GM through medication, diet, and exercise has improved the symptoms of T2DM, what role do specific genera play in this process? At what level of abundance and number of these genera is optimal? Limited by current technology, we are unable to directly assay genera directly in the human body. These are questions that current researchers need to overcome.

### 4.3 Current hot spots and development prediction of GM and T2DM-related studies

We explore current research themes based on studies conducted by influential scholars, Fukui Michiaki and Hamaguchi Masahide, who have co-authored a number of research studies focusing on the improvement of GM and T2DM with diet and exercise, and further exploring specific mechanisms of action (54–58). Burcelin and Remy’s researches focus on “brain-gut interactions”, but they also focus on the effects of pharmacological interventions on GM and the specific mechanisms involved (14, 39, 59–61). Several other high-impact scholars have focused on the same topic, addressing the effects of diet, exercise, and medications on GM and T2DM (62–67).

In terms of keywords, in addition to gut microbiota and type 2 diabetes mellitus, obesity, insulin resistance, inflammation, and other related symptoms and diseases have been the focus of scholars. This suggests that current research is focused on exploring the link between GM and T2DM and its complications. The 18 significant clusters in the results of the cluster analysis relate to the content of three topics: (1) Diabetes mellitus and its physiological processes. (2) GM and methods of regulating GM. (3) Types of studies, experimental techniques, and testing metrics. These three topics are important components for conducting relevant research.

Combined with the results of the above analysis, future research may have the following trends: (1) Future research will not only focus on T2DM itself, but will also expand to other T2DM-related diseases or specific physiological processes. (2) The way to regulate GM will be further refined, and the specific mechanisms of exercise, diet, and drug regulation of GM will become the focus of research. (3) New experimental techniques and detection indexes will be applied in related studies to help form more convincing research conclusions.

## 5 Conclusion

In this study, we systematically sorted out and quantitatively analyzed the relevant studies on regulating GM to improve T2DM, demonstrating the current research status and future research trends in this field. The results of this study can help researchers gain a comprehensive understanding of the current state of research and provide a reference for future researches. For researchers, future studies will delve more deeply into the specific mechanisms that regulate GM to ameliorate T2DM and will extend to T2DM-related diseases and symptoms. Therefore, the development of new experimental techniques and new assays is necessary. For clinicians, further optimization of GM interventions and development of individualized therapeutic strategies for patients with T2DM may lead to better clinical outcomes.

## 6 Strengths and limitations of this study

In this study we searched only publications in the WOSCC database and limited the language of the publications to English. Therefore, there may be omissions in the included publications. In addition, although we considered all possible search terms, we still could not guarantee that there were no omissions in the search results. In conclusion, our study is sufficiently precise but may not be complete.

## Author contributions

RJ: Conceptualization, Writing – original draft. ZC: Data curation, Formal analysis, Writing – original draft. LiZ: Conceptualization, Methodology, Writing – original draft. LoZ: Validation, Writing – review & editing. QG: Data curation, Writing – review & editing. SW: Formal analysis, Writing – original draft. JF: Investigation, Methodology, Writing – review & editing. JC: Writing – original draft. ML: Supervision, Writing – review & editing.

## Supplementary material

Supplementary Figure S1 Keyword timeline analysis view.
