# Supplementary figures and images for: Global research trends in regulating gut microbiome to improve type 2 diabetes mellitus: bibliometrics and visual analysis
Source: Front Endocrinol (Lausanne). 2024 Jun 3;15:1401070. doi: 10.3389/fendo.2024.1401070 (PMC11181692; doi:10.3389/fendo.2024.1401070)

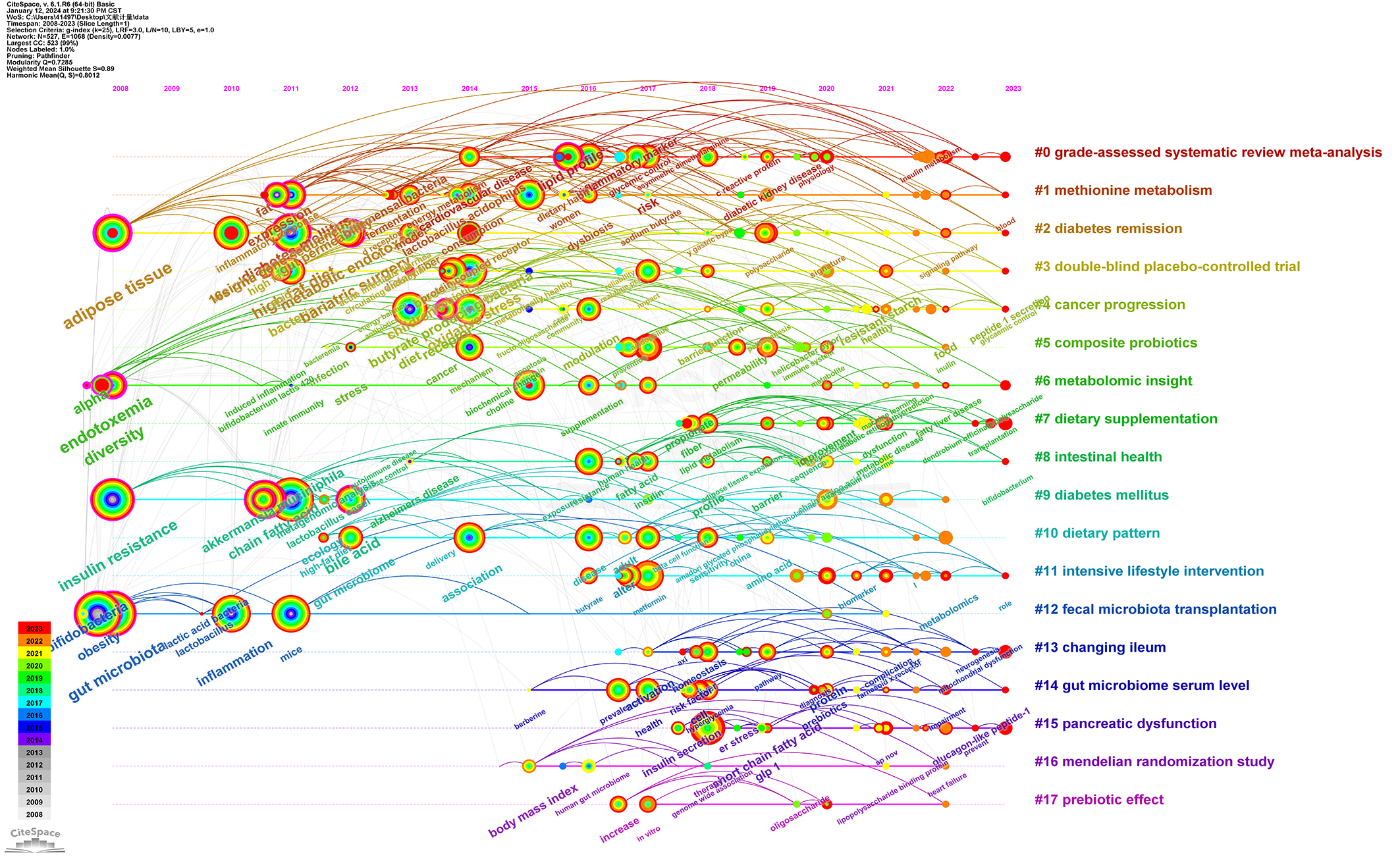

Supplement: Supplementary Figure S1 — Keyword timeline analysis view. [file Image_1.tif]
